# The Influence of *Lactobacillus* spp. Secondary Metabolites Isolated from Immature Egyptian Honey on Human Pathogens, Transcription of Virulence Genes and Lung Cancer

**DOI:** 10.1007/s12088-024-01224-7

**Published:** 2024-03-04

**Authors:** Tarek R. Elsayed, Eman Nour, Ahmed A. Hamed, Ashwak Abdel-Moneim Hassan, Yasser Essam Elenany

**Affiliations:** 1 Department of Agricultural Microbiology, Faculty of Agriculture, Cairo University, Giza, 12613 Egypt; 2 Faculty of Organic Agriculture, Heliopolis University for Sustainable Development, Cairo, 11785 Egypt; 3 Pharmacognosy Department, Faculty of Pharmacy, Cairo University, Kasrel Aini St., Cairo, 11562 Egypt; 4 Department of Dairy Science, Faculty of Agriculture, Cairo University, Giza, 12613 Egypt; 5 Department of Economic Entomology and Pesticides, Faculty of Agriculture, Cairo University, 3 El Gamaa St., Giza, 12613 Egypt

**Keywords:** Egyptian immature honey, Lactic acid bacteria, Molecular identification, Cytotoxicity, Antagonistic activity, HPLC-QTOF

## Abstract

This work aimed to isolate, and identify Lactic Acid Bacteria LAB from Egyptian immature citrus honey, and characterize their secondary metabolites, as well as determine the antibacterial activities and transcription of virulence genes (stx1, stx2, and eae) influenced by these bacterial secondary metabolites. From twenty hives, twenty immature citrus bee honey samples were taken. Traditional cultural and biochemical testing were used, followed by molecular confirmation. Further, LAB isolates' antibacterial and cytotoxic properties were investigated. 16S rRNA gene sequencing were assessed and, two lactic acid bacterial isolates were identified as *Lactobacillus acidophilus* Ch_2_ and *Levilactobacillus brevis* Ch_1_. Both isolates have good antagonistic action against clinical pathogens, with *Levilactobacillus brevis* Ch_1_ exhibiting the best antibacterial activity against all indicator pathogens examined. When compared to untreated cancer cells, the isolates demonstrated significant cytotoxic activity. Ch_1_ and Ch_2_ cell viability percentages were 39.5% and 18.76%, respectively. Furthermore, when exposed to *Levilactobacillus brevis* Ch_1_ metabolites, Shiga-producing *Escherichia coli* (STEC) virulence gene expression was suppressed. To identify bacterial secondary metabolites, a high-performance liquid chromatography-quadrupole time-of-flight mass spectrometry (HPLC-QTOF) approach was developed. Twenty-seven metabolites from diverse chemical classes were discovered in the crude extracts with antibacterial and anticancer characteristics. This is the first thorough investigation on the metabolic profile of LAB isolated from immature Egyptian honey and the findings suggested that isolates or their secondary metabolites could be used in the food sector as medicinal alternatives or as a biocontrol agent.

## Introduction

Bees use plant nectar, plant secretions, or honeydew produced by insects that consume plants to make honey (*Hemiptera*). Foraging bees mix this raw material with their secretions to convert it before maturing it into honeycomb cells. As honey ages, its biotransformation, biodegradability, and the bioaccumulation cause change to its properties, chemical compositions, and microbiological content [1]. The authenticity and quality of natural honey are greatly affected by these modifications. Also, these immature honey features, particularly its higher moisture content than honeybee honey, will ultimately give rise bacterial fermentation of immature honey [1]. Furthermore, the warm, humid environment of a beehive is ideal for some bacteria to flourish quickly. Lactic acid bacteria present during the fermentation process enrich the immature honey with microorganisms like lactic acid bacteria (LAB), some of which have probiotic qualities [2]. Lactic acid bacteria can be found in nutrient-rich environments such as those found in people, animals, food, and plants [3]. The majority of study on the microbes associated with honey has been conducted with the goal of discovering and isolating the beneficial bacteria associated with honey. On the other hand, little is known about the cytotoxicity of LAB isolated from bee honey and honeybees, neither about the antibacterial qualities nor the transcription of human pathogen genes. Lactic acid bacteria are being employed more and more in both humans and animals, especially for the treatment and prevention of a variety of illnesses and conditions like cancer, diabetes, allergies, hypertension, obesity, and immune system enhancement [4]. It is worth noting that different LAB strains utilized in the fermented food sector have different metabolic capabilities, resulting in a variety of bioactive components [4]. Metabolites are small molecular weight molecules (> 2500 amu) that result from a metabolic reaction as an intermediate or end product. Bacterial metabolites are classified into two types: primary and secondary metabolites, which are produced at distinct stages of development [5]. Primary metabolites are necessary for the development of microorganisms and are created during the log phase of growth. Several primary metabolites are important in industry and are produced on a large scale by fermentation. The Primary metabolites, on the other hand, have no pharmacological actions or effects. As a result, it varies from secondary metabolites, which have a wide range of significant and effective actions [6]. During the stationary phase, secondary metabolites are generated. Antibiotics, enzyme inhibitors, growth promoters, and other pharmacologically important substances that are not required for growth but have a variety of industrial applications are examples of metabolites. Recently, a metabolomic technique has been employed to detect metabolites for secondary metabolites [7, 8]. As a result, we designed a study to isolate and identify lactic acid bacteria from Egyptian immature Citrus bee honey using 16S rRNA sequencing, as well as study their antagonistic and anticancer activities and identify metabolites using high-performance liquid chromatography-quadrupole time-of-flight mass spectrometry (HPLC-QTOF). The long-term goal would be to utilize beneficial microbes or their products as an additional and sustainable technique for disease control or as a natural preservative in fermented food manufacturing.

## Materials and Methods

### Honey Sampling

Twenty samples of immature Citrus bee honey were used in this study, taken from twenty bee hive combs, ten were in apiaries in the Kafr El-Sheikh governorate and ten in the Faculty of Agriculture at Cairo University. As reported by [9], all sample were taken in an aseptic manner with a sterile 5 mL syringe and stored in a twenty mL sterile dark glass recipient. The samples were immediately transferred for microbiological evaluation.

### Isolation of LAB

LAB was isolated using a slightly modified version of the procedure outlined by Hasali et al. [9]. A sterile glass container containing 90 ml of peptone saline solution was filled with 10 g of immature citrus honey, to create a first dilution, the contents of the glass were mixed and homogenized. Serial dilutions were produced for each sample, and 1 mL of the appropriate dilution was then combined with the melted MRS (Difco Laboratories, India) agar. The MRS plates were treated with cycloheximide 0.01% (v/v) to inhibit the development of fungi. After incubation at 37 °C in 5% carbon dioxide incubator for 48 h colonies with unique morphological characteristics were chosen and then purified.

### LAB Preliminary Characterization

Gramm staining, cell morphology, catalase and oxidase activity, milk coagulation, and gas generation from glucose in MRS broth, as previously described by Hassan et al. [10], were utilized to differentiate the presumed LAB isolates. The non-LAB isolates were discarded under strict safety conditions. Gram-positive isolates showed negative catalase and oxidase activity were stored at − 20 °C in MRS broth with 15% glycerol added.

### Scanning Electron Microscope Visualization

Bacterial cells suspensions were centrifuged at 3000 × *g* for 5 min. Samples of pellets were prepared and examined at 10–25 kV through Scanning Electron Microscope as previously described by [11]. Scanning Prop Image Processor (SPIP) program Software for Microscopy Image Analysis were used.

### Molecular Identification of Lactic Acid Bacteria

Polymerase chain reaction (PCR) and DNA sequencing of the 16S rRNA gene were performed in accordance with the procedure provided by Ahmed et al. [12]. Bacterial cells were extracted in a microcentrifuge tube after being centrifuged for 10 min at 5000 × *g*. The pellets were washing for three times using 0.85% NaCl solution, genomic DNA was extracted using Gene JET Genomic DNA purification Kit (Thermo Fisher Scientific, Republic of Lithuania). A set of bacterial universal primers F-27 (5′–AGAGTT TGATCMTGGCTCAG–3′) and R-1494 (5′–CTACGG YTACCTTGTTACGAC–3′) were used for amplification step as described by [12]. Polymerase Chain Reaction (PCR) was carried out using Master cycler (Eppendorf, Homburg, Germany) and the amplification step was done using Thermal cycler PCR (Bio-rad T100, USA). The PCR products with expected size of 1.5 kb were inspected with ethidium bromide-stained agarose, visualized and photographed. the PCR products were purified using a QIA quick PCR purification kit (QIAGEN, Hilden, Germany) as described by the manufacturer’s protocol. The purified PCR products were then sequenced by Macrogen, Inc., Seoul, South Korea using automatic ABI 370 × 1 DNA Sequencer (Applied Biosystem, USA). Databases were examined for similarity by using the BLAST programme (National Center for Biotechnology Information NCBI, USA). A Phylogenetic tree was constructed based on 16S rRNA gene sequences using MEGA x 11software (http://www.megasoftware.net/mega7). The isolates' relationship to other closely similar NCBI GenBank reference taxonomy sequences was depicted as a tree.

### Antagonistic Activity of Isolated LAB

The antibacterial activity of identified isolates against human pathogens was assessed using the agar well diffusion technique put out by five Hassan et al. [10] with light modification. In brief, the isolates were inoculated in MRS broth and incubated at 37 °C for 24 h before being collected by centrifugation (6000 g for 15 min at 4 °C). Cell-free supernatants were adjusted to pH 7.0 with 1N NaOH, sterilized with 0.45 m filters (Sartorius, Germany), and referred to as “crude extracts”. The selected human pathogens comprised *Escherichia coli* O157H:7, Methicillin-resistant *Staphylococcus aureus* (MRSA)*, Bacillus cereus* ATCC 33018, *Salmonella typhimurium* ATCC 14028 and *Pseudomonas aeruginosa*. Inhibition zones around wells was measured and interpreted following [13] method.

### Cytotoxicity Test

The ATCC (American Type Culture Collection., USA) provided human NSCLC cell lines A549, which were cultured at 37 °C in 5% CO2 RPMI media fortified with 10% fetal bovine serum (FS; HyClone Laboratories, Inc., USA) and a penicillin/streptomycin solution of 10,000 U/ml (Sigma-Aldrich., USA). In vitro tissue culture study on the human lung cancer cell line (A549) using neutral red dye were performed to test the effect of isolates secondary metabolites on the viability of the cancer cell line followed the method described by El-Deeb et al. [14]. According to the previous study [13], the stain intensity was measured using a Spectrofluorometer at 540 nm, and the anti-cancer activity of the samples was evaluated using a neutral red uptake assay. The cell viability was estimated using the following equation:

$${\text{Viability}}\;\% = {\text{Absorbance of sample}}/{\text{Absorbance of control}} \times 100.$$

### RNA Extraction and cDNA Synthesis

*Escherichia coli* O157H: was grown in Luria–Bertani (LB) broth at 37 °C for 24 h. This strain of bacteria produces the Shiga toxins Stx1, Stx2, and eae. Prior to RNA extraction, bacterial cells were centrifuged to concentrate them. Following sample collection, 8 mL (2 volumes) of RNA Protect Bacteria Reagent (Qiagen) was added to each 4 mL sample. Before extracting the RNA, the samples were centrifuged for 10 min at 5000 × *g*, and the supernatants were then drained. employing the RNeasy Mini Kit (Qiagen, Mississauga, ON, Canada), RNA was extracted for Gram-negative bacteria in accordance with the manufacturer's instructions. The Turbo DNA-freeTM kit (Ambion, Cambridge, UK) was used to remove contaminated genomic DNA from each RNA preparation in accordance with the DNase treatment recommendations provided by the manufacturer. Utilizing a Thermo Scientific Nanodrop 2000 (ON, Canada), the concentration of total RNA was ascertained. Then, following the manufacturer's instructions, 0.3 mg of RNA was processed in reverse using Superscript III. (Invitrogen, Carlsbad, CA, USA) [15].

### Real-Time PCR

Using a comparative real-time PCR, the transcription of the virulence genes (stx1, stx2, and eae) was assessed. One milliliter of reverse-transcribed cDNA, twelve and a half milliliters of Power SYBRR Green PCR Master Mix, 0.25 units of AmpEraseR Uracil N-Glycosylase (UNG; Applied Biosystems), two milliliters of each primer, and six milliliters of nuclease-free water were added. Every primer utilized in the investigation has been previously documented [16].

### Extraction of Secondary Metabolites from Isolates

According to the method described by [17] briefly, after adding equal parts of cell-free culture broth and ethyl acetate to a reparatory funnel and shaking the mixture briskly for half an hour, the ethyl acetate portion was collected. Three times through the process, the material was pooled and dried at 45 °C using a rotary evaporator. A milliliter of methanol was used to reconstitute the crude extract. Samples were gathered in HPLC glass vials and filtered through an ultra-membrane filter (pore size 0.45 µm) for spectrum analysis.

### Liquid Chromatography with Tandem Mass Spectrometry (LC–MS/MS) Analysis

Using a high-performance liquid chromatography quadrupole time-of-flight mass spectrometry device, the crude ethyl acetate extracts were examined. The HPLC-QT oF analysis was preformed using an Agilent 6530 HPLC equipped with a Zorbax Extend-C18 RRHD 2.7 µm column measuring 3 × 150 mm. Elution was performed using 0.5 mL/min of isocratic 90% H2O/MeCN for one minute, and then a gradient elution with isocratic 0.1% formic acid modifier to 100% MeCN over 30 min. Using an Agilent 6530 Q-ToF, aliquots (1 µL) of test solutions containing 100 µg/mL of analyte in 100 µL MeOH were examined. For ions found in the whole scan at an intensity over 1000 counts at 10 scans/s, with an isolation width of 4 ~ m/z, a fixed collision energy (20 eV), and a maximum of 3 selected precursors per cycle, MS/MS analysis was carried out on the same instrument. Using the ProteoWizard software program Msconvert, the mass spectrometry data acquired during LC–MS/MS analysis have been transformed to the mzXML file format [15].

### MS/MS Data Pre-processing

MZmine 2.53 was used to parse the mzXML files and produce feature peak lists. Table 1 provides detail on the relevant variables [17]. The characteristics corresponding to methanol and blank media were eliminated.

**Table 1:** Parameters for MZmine processing of HPLC–MS/MS data

Processing step	Parameter	Selected values
Mass list	MS1 noise level	5E3
	MS2 noise level	1E2
	Rt (retention time in minutes)	0–30 min
Chromatogram building	Algorithm	ADAP chromatogram builder
	Min group size in number of scans	5
	Group intensity threshold	5E3
	Min highest intensity	1E4
	m/z tolerance	20 ppm
Deconvolution	Algorithm	Local minimal search
	Chromatographic threshold	90%
	Search minimum in RT range	0.15 min
	Minimum relative height	0.001%
	Minimum absolute height	5E3
	Min ratio of peak top/edge	1.3
	Peak duration range	0.01–5 min
Isotope grouping	m/z tolerance	10 ppm
	RT tolerance	0.5
	Maximum charge	2
Alignment	Algorithm	Join aligner
	m/z tolerance	20 ppm
	Weight for m/z	75%
	RT tolerance	0.5 min
	Weight for RT	25%

### Statistical analysis

Analysis of variance (ANOVA) was used to statistically analyse the results of triplicate samples using MSTAT-C software. The data is presented as the three replicates' means ± standard deviations. When *P* ≤ 0.05, differences in means are considered statistically significant.

## Results and Discussion

### Isolation and Identification of LAB

**Fig. 1:**
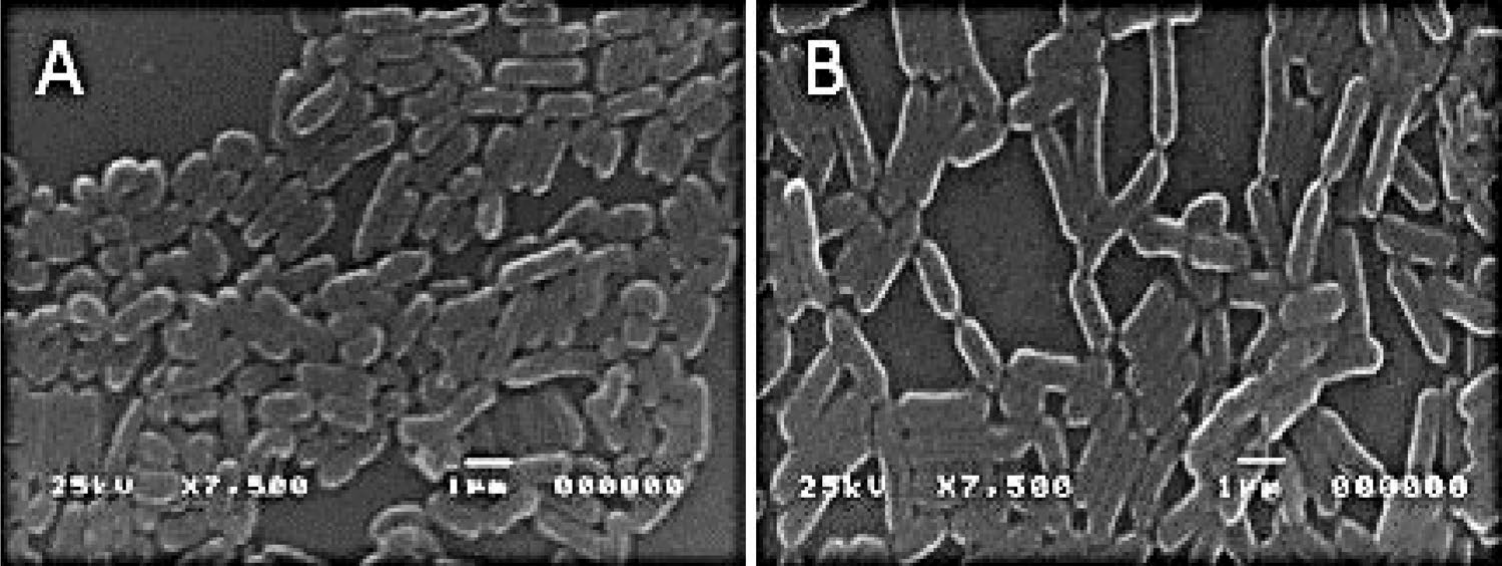
Scanning electron micrographs (7500 ×) of bacterial cell isolated **A** Ch_2_ and **B** Ch_1_ from immature Egyptian citrus honey. Scale bar is 10 µm

Twenty immature citrus bee honey samples yielded thirty-four isolates of bacteria and yeasts in this study. Two unique isolates (Ch_1_, and Ch_2_) with different morphologies were chosen for further study (Table 2). After 24 h in MRS Microaerophiles agar media, isolate Ch_1_ developed a greyish-white, opaque, and convex colony, whereas isolate Ch_2_ developed a beige colony with a spherical, smooth morphology. Gram-positive, oxidase- and catalase-negative isolates met the LAB selection criteria in Bergey's Manual of Determinative Bacteriology [18, 19]. Isolates showed no textural defects, such as gas bubbles, and have good coagulation properties. In the Ch_2_ isolate, milk coagulated after 10 h, but in the Ch_1_ isolate it took 14 h. The pH of the blank was 6.58, but the pH of Ch_1_ and Ch_2_ were 4.58 and 4.60, respectively. Neither of the two isolates created carbon dioxide [20]. Further, SEM micrographs revealed that bacterial isolates were rod-shaped (Fig. 1). It is significant to realize that the curved geometries of numerous cells prevented their length from being determined. For individuals with a straight cell and good sight, the length varied greatly, ranging from 1.03 ± 0.04 for Ch_2_ to 1.53 ± 0.19 µm for Ch_1_ (Table 2). The average cell width for Ch_2_ was 0.85 ± 0.02 µm, whereas it was 1.07 ± 0.02 µm for Ch_1_. According to Young's [21] investigation, *Lactobacillus delbrueckii* cells measure between 0.5 and 0.8 µm in width and 2 and 9 µm in length. These findings are in line with our findings. Elongation is a measurement that indicates how long a form is. A square or circle will always return zero. As the form changes to a long rectangle or an ellipse, the returned value approaches 1.0. In terms of isolates, the elongation ranges between 0.552 ± 0.02Ch_1_ and 0.783 ± 0.04 m Ch_2_. The disparities in cell elongation can be explained in part by the fact that, with few exceptions, immature cells are substantially longer than old or mature cells.

**Table 2:** Phenotypic characterization of presumptive lactic acid bacteria isolated from immature Citrus honey

Bacterial strains	Gram staining	Catalase and oxidase tests	pH	Coagulation times hr	Colony morphology	Cell morphology	Length	Width	Elongation
							µm		
Ch_1_	+	−	4.58	14	Greyish-white, pinpoint, colony, convex elevation, smooth-edged	Slender rods non motile, non-sporulated form chains	1.53 ± 0.19	1.07 ± 0.02	0.552 ± 0.02
Ch_2_	+	−	4.60	10	White big colonies, rounded, smooth	Rods non motile, non-sporulated form chains	1.03 ± 0.04	0.85 ± 0.02	0.783 ± 0.04

Data means ± SD (n = 3)

**Fig. 2:**
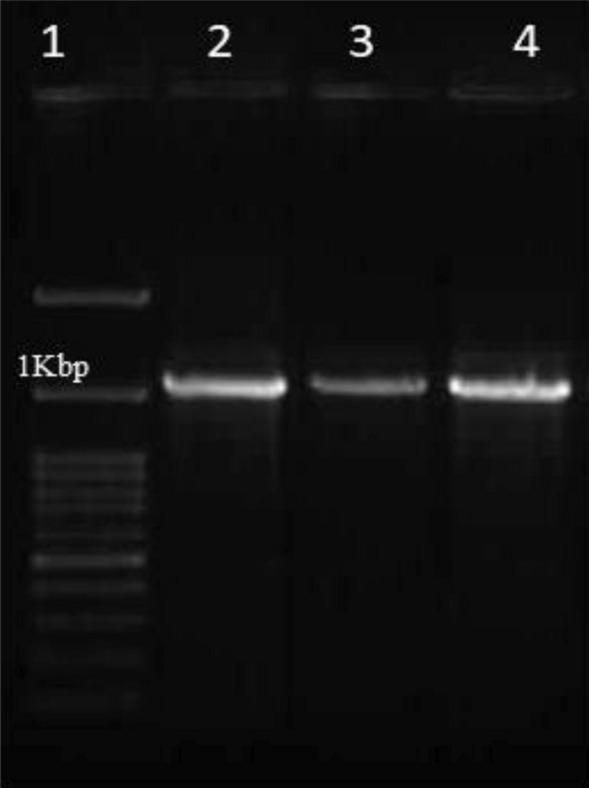
A Photo illustrating the detected PCR products on gel electrophoresis for amplified segments of the16S rRNA gene. Lane 1: 1Kbp ladder, lane 2, 3: amplicons of 16S rRNA gene produced by isolates *Levilactobacillus brevis* MT611665.1 and *Lactobacillus acidophilus* MT515942.1, respectively, lane 4: negative control

**Fig. 3:**
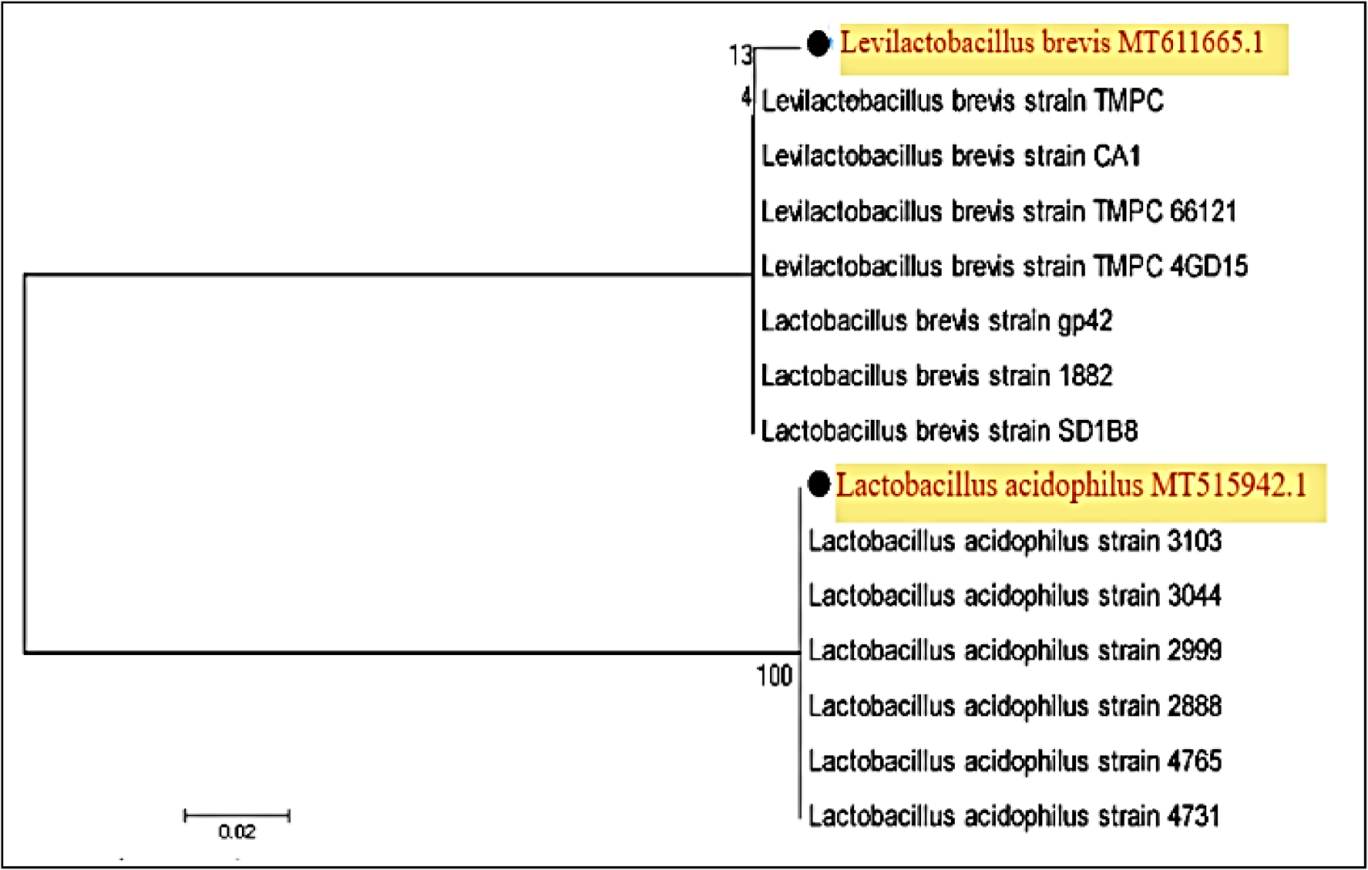
Neighbor-joining phylogenetic tree (http://www.megasoftware.net/mega7) based on 16S rRNA gene sequences of LAB isolates (dark circles) with the closest hits obtained from the NCBI GenBank

Following phenotypic description, partial 16S rRNA sequence analysis was done to identify the two selected isolates using two universal primers [22]. Every isolate chosen produced a 1500 bp PCR output (Fig. 2.). These PCR products were purified with the QIA quick PCR Purification Kit, and the resulting DNA was sequenced. The identities of all isolates were sufficiently verified by sequence analysis (BLAST) after scanning the GenBank database (NCBI, USA) for 16S rRNA sequence similarities. The isolates Ch_1_ and Ch_2_ had 99.86% and 98.16% similarity to *Levilactobacillus brevis* MT611665.1 and *Lactobacillus acidophilus* MT515942.1, respectively, based on the 16S rRNA sequence (Table 3). Figure 3 depicts the phylogenetic analysis utilizing the closest hits found by reconstructing a phylogenetic tree from the NCBI Gene Bank. Aween et al. [23] also identified *Lactobacillus acidophilus* from honey marketed commercially in Malaysia. Lashani et al. [24] isolated *L. paracasei*, *L. brevis, L. rhamnoses*, and *L. fermentum* from honey in an earlier study.

**Table 3:** List of lactic acid bacteria isolated from Egyptian immature citrus honey and their GenBank accession numbers acquired based on 16S rRNA gene sequencing

16S rRNA	GenBank accession number	Sequence lengths (bp)	Max. Ident. %
*Levilactobacillus brevis*	MT611665.1	221	99.86
*Lactobacillus acidophilus*	MT515942.1	913	98.16

### Antagonistic Activity

**Fig. 4:**
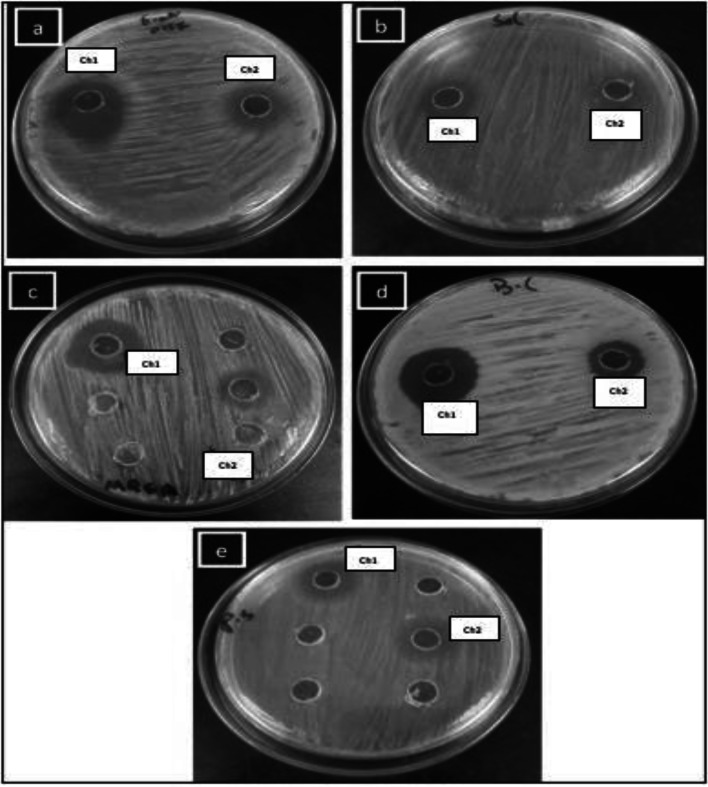
The inhibitory effect of *Levilactobacillus brevis* Ch_1_ and *Lactobacillus acidophilus*Ch_2_ against *Escherichia coli* O157H:7 (**a**), *Salmonella typhimurium* (ATCC14029) (**b**), *Staphylococcus aureus* MRSA (**c**), *Bacillus cereus* ATCC 33018 (**d**) and *Pseudomonas aeruginosa* ATCC 9027 (**e**)

The antibacterial activity of Lactobacillus sp. crude extracts against the aforementioned pathogenic microorganisms was determined using the agar well diffusion method. Table 4 shows the antibacterial activity data in terms of zone of inhibition (ZOI) diameter. A diameter of more than 1 mm surrounding the well was considered a positive result. The higher the width of the ZOI, the better the antibacterial activity of the isolate. *Lactobacillus* sp. exhibited substantial (*p* 0.05) antagonistic responses against all pathogenic strains tested. The results show that the antibacterial capability of *Levilactobacillus brevis* Ch_1_ was the strongest against all of the indicator pathogens tested (Fig. 4). It was most effective against *B. cereus*, MRSA, and *E. coli*, with ZOIs of 7 ± 1.3, 6 ± 1.5, and 6 ± 1. 4 mm, respectively, and was not as effective against *Pseudomonas aeruginosa* (3 ± 1.1 mm). Furthermore, *Lactobacillus acidophilus* Ch_2_ demonstrated antimicrobial action against all pathogens tested, with the maximum activity against *B. cereus* (4 ± 1.1) and the lowest activity against *E. coli* (1 ± 1.3 mm). *Pseudomonas aeruginosa* was equally inhibited by *Levilactobacillus brevis* Ch_1_ and *Lactobacillus acidophilus* Ch_2_. Our findings corroborated prior research [25–28] that revealed the possible release of LAB bioactive components such as organic acids, hydrogen peroxide, and bacteriocins [25], which inhibit pathogen growth. *Levilactobacillus brevis* Ch_1_ inhibited tested harmful bacteria, demonstrating their potential as bio-therapeutic mediators for enhancing digestive tract health in humans. More research focusing on using Ch_1_ as a therapeutic agent may prove advantageous and broaden their therapeutic repertory for clinical applications.

**Table 4:** Antagonistic activity of tested cell free crude extract

Pathogenic microorganisms	Inhibition zone (mm) (means ± SD)	
	*Levilactobacillus brevis* Ch_1_	*Lactobacillus acidophilus* Ch_2_
*E. coli* O157H:7	6 ± 1.4^a^	1 ± 1.3^b^
*Salmonella typhimurium* ATCC14028	4 ± 1.1^a^	2 ± 0.5^b^
*Staphylococcus aureus* MRSA ATCC43300	6 ± 1.5^a^	3 ± 0.3^b^
*Bacillus cereus* ATCC 33018	7 ± 1.3^a^	4 ± 1.1^b^
*Pseudomonas aeruginosa* ATCC 9027	3 ± 1.0^a^	3 ± 1.0^a^

Data are means ± SD (n = 3). Different superscript letters in the same row indicate significant difference (*p* < 0.05)

### Cytotoxicity Screening

**Fig. 5:**
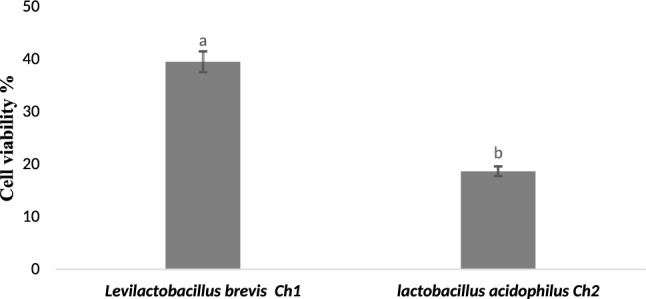
Cytotoxicity Assessments in A-549 Cells Following the Exposure of *lactobacillus* sp. Secondary metabolites for 24 h. Values are mean ± SD of three independent experiments

**Fig. 6:**
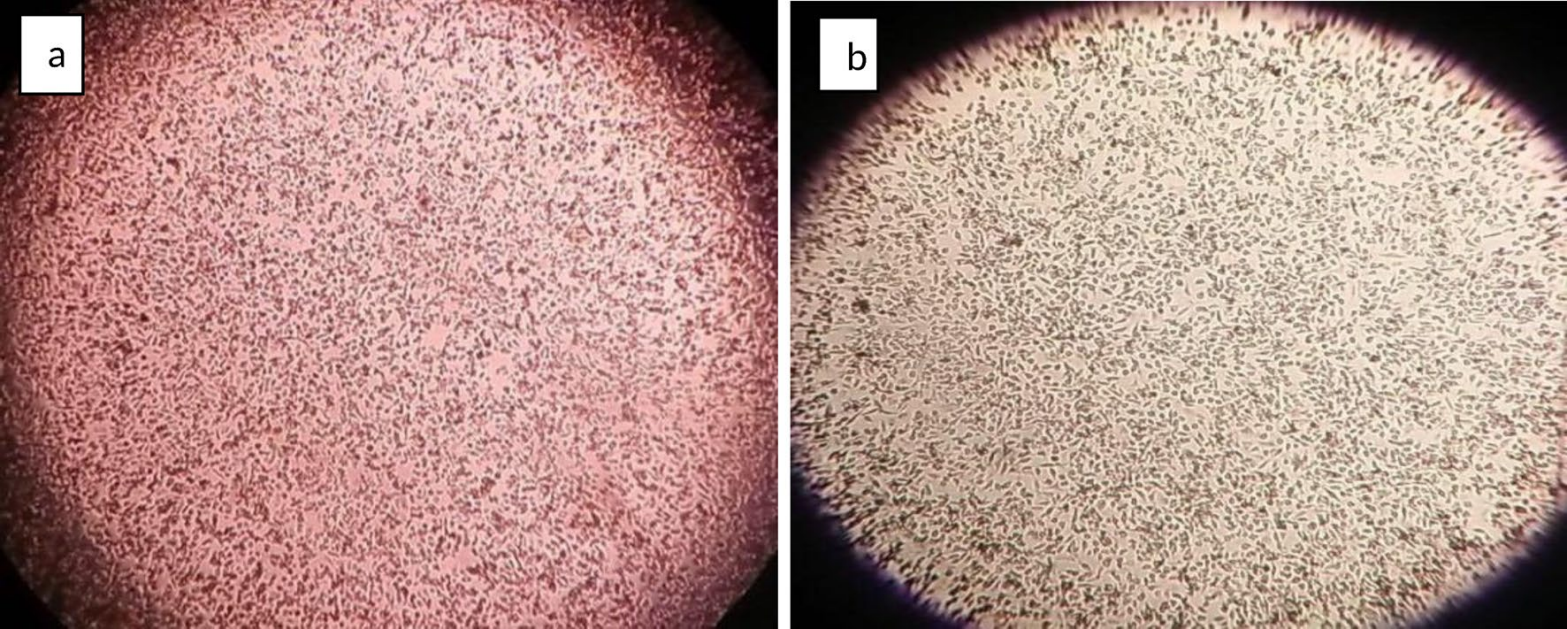
Anti-cancer effect of *Levilactobacillus brevis* Ch_1_ (**a**) and *Lactobacillus acidophilus* Ch_2_ (**b**) on the lung cancer cell line (A549) under microscope

Following a 24-hour exposure of the A-549 cells, the cytotoxicity of the secondary metabolites of *Lactobacillus acidophilus* Ch_2_ and *Levilactobacillus brevis* Ch_1_ was evaluated. Figure 5 shows the percentage of A-549 cells that are viable. The findings indicate that the secondary metabolites of *Lactobacillus* sp. caused a statistically significant (*p* < 0.05) reduction in A-549 cell viability. The percentages of cell viability for *Lactobacillus acidophilus* Ch_2_ and *Levilactobacillus brevis* Ch_1_ were 18.76% and 39.5%, respectively. Under a microscope, Fig. 6 demonstrated lactobacillus sp. anti-cancer impact on the lung cancer cell line A-549. A malignant tumor with a high incidence and fatality rate worldwide is lung cancer. Probiotics have been shown to have anti-tumor properties in recent years, and *Lactobacillus* is one of the most extensively researched probiotics used in the treatment of lung cancer. According to Espirito et al. [29], *Lactobacillus* sp. can stop tumor cells from metastasizing to the lung. Members of the *Lactobacillus* genus create compounds that can alter a host's respiratory immunity. Propionate, butyrate, and acetate are among the SCFAs that have the greatest impact on pulmonary immunity from gut microbes. Our findings are consistent with Zhang et al. [30], who reported that *L. casei* effectively inhibits the proliferation of A549 lung cancer cells in vitro (strain not stated). Additionally, probiotic capsules (mostly made of *Lactobacillus* species) added to the gut greatly lower the risk of lung deterioration and improves the quality of life in lung cancer patients as mentioned by Du et al. [31].

### Effect of ***Levilactobacillus brevis*** Ch_1_ on ***E. coli*** O157:H7 Stx1 Stx2 and Eae Gene Expression

**Fig. 7:**
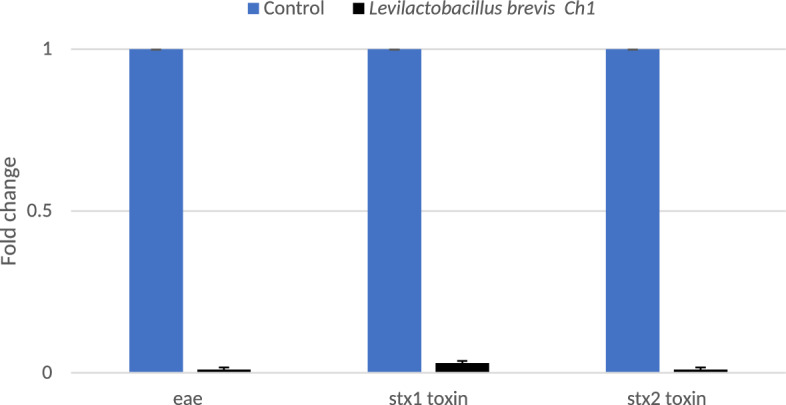
Effect of *Lactobacillus brevis* Ch1 secondary metabolites on gene expression of *E. coli* O157:H7. Relative gene transcription shows the change in (control, value of 1.0). The transcription of each gene was contrasted to the 16S rRNA transcription in each sample. Data are presented as the means ± SE for RNA isolated from three replicates

The influence of *Levilactobacillus brevis* Ch_1_ secondary metabolites on virulence gene transcription of *E. coli* O157:H7 was investigated as a result of potent inhibitory effect of Ch_1_ against cancer cell and tested pathogens. Well-known virulence genes were the focus of our investigation, including the Stx genes stx1 and stx2, and the genes producing intimin (eae) (Fig. 7). The expression of the eae gene was considerably (*P* < 0.05) downregulated following exposure to the secondary metabolites of *Levilactobacillus brevis* Ch_2_. According to Franz et al. [32], the presence of the eae gene is substantially associated with the abundance of genes encoding a number of various virulence factors. The virulence factor of O157:H7 that cannot be disputed is the production of Stx [32–35]. O157:H7 generates two types of Stx: Stx1 and Stx2. Stx1 has three subtypes (a, c, and d), while Stx2 has seven (a, b, c, d, e, f, and g). Although both toxins may produce bloody diarrhea and HUS, a specific subset of stx2 subtypes (stx2a, stx2c, and stx2d) show a stronger correlation with HC and HUS than stx1 subtypes or other stx2 subtypes [34]. In this work, exposure to *Levilactobacillus brevis* Ch_1_ secondary metabolites resulted in a significant downregulation of the stx1 and stx2 genes. Similar results were achieved by [36], who reported that *L. acidophilus* La-5 secretes a molecule or molecules that limit transcription of EHEC O157 genes implicated in colonization by either acting as a QS signal inhibitor or directly interacting with bacterial transcriptional regulators.

### Dereplication of Metabolites via HPLC-ESI-QTOF-MS/MS

Since germs are growing resistant to different medicines, it is vital to find new treatments to treat diverse infectious diseases [37]. Nowadays, researchers are searching for the habitat of honeybees for novel medicinal microbes. For this experiment, we have selected two different antagonistic bacteria that are active against human diseases. Table 5 shows how lactobacillus sp. Produces secondary metabolites, which is one of the ways that bacteria protect their hosts from various infections and predators [38]. The bacterial flora associated with honeys has the capacity to generate several bioactive compounds, which can be detected using HPLC-ESI-QTOF-MS/MS analysis. After conducting a chemical study, 27 metabolites were found to be associated with various chemical classes, including steroids, phospholipids, hopanoids, triglycerides, and derivatives of alkylamines. Additionally, as reported by Chiba et al. [39], the investigation revealed the existence of several classes of secondary metabolites with antibacterial and anticancer characteristics, including Medelamine A and Rhodopeptin B5, a new cyclic tetrapeptide with antifungal capabilities like7,8-Didehydro-3-methylprostreptovaricin, N-isopentyltridecanamide, and 3'''-Adenylylspectinomycin. In order to create comprehensive metabolic profiles of different lactic acid bacterial genera and species, the current dereplication and quantification technique can be expanded upon. This could be helpful in the field of finding drugs from beneficial bacteria. Furthermore, our findings suggest that the selected LAB strains may be valuable sources for the production of probiotics and functional foods enhanced with anticancer properties.

**Table 5:** Secondary metabolites putatively annotated in *Levilactobacillus brevis* Ch_1_ and *lactobacillus acidophilus* Ch_2_ through HPLC-HRMS analysis

No	t_R_ (min)	Compound	Molecular ion	Adduct	Molecular formula	Occurrence
1	1.7	Double oxidized cysteine	174.992	M + Na	C3H6NO4S	Ch_1_
2	9.49	6-tert-Butyl-2-cyclopentylphenol	219.175	M + H	C15H22O	Ch_2_
3	11.34	(z)-4-(hydroxycarbonyl)-2-amino-4-hydroxybutanoic acid	298.041	M + K	C9H13N3O6	Ch_1_ and Ch_2_
4	11.7	Medelamine A	214.253	M + H	C14H31N	Ch_2_
5	14.37	(2R,3S,4S,5R,6S)-2-(hydroxymethyl)-6-(4-hydroxyphenyl) sulfanyloxane-3,4,5-triol	289.0752	M + H	C12H16O6S	Ch_2_
6	16.7	(8S,9S,10R,13R,14S,17R)-10,13-Dimethyl-17-[(2R)-6-methylheptan-2-yl]-2,3,6,7,8,9,11,12,14,15,16,17-dodecahydro-1H-cyclopenta[a]phenanthren-3-amine	386.3811	M + H	C27H47N	Ch_1_
7	16.78	(3β,4α,5α)-4,4-Dimethylcholesta-8(14),24-dien-3-OL	413.379	M + H	C29H48O	Ch_2_
8	17.05	N-undecylundecan-1-amine	326.3778	M + H	C22H47N	Ch_2_ and Ch_1_
9	17.14	Rhodopeptin B5	546.3997	M + Na	C28H53N5O4	Ch_2_
10	17.15	N-[(E,2S,3R)-1,3-dihydroxyhexadec-4-en-2-yl]docosanamide	594.5786	M + H	C38H75NO3	Ch_1_ and Ch_2_
11	17.21	(2R)-2-hydroxy-N-[(2R,3R,4E,8E)-3-hydroxy-1-[(2R,3R,4S,5S,6R)-3,4,5-trihydroxy-6-(hydroxymethyl)oxan-2-yl]oxyoctadeca-4,8-dien-2-yl]tetradecanamide	708.5103	M + Na	C38H71NO9	Ch_2_
12	17.37	bacteriohopanetetrol cyclitol ether	708.514	M + H	C41H73NO8	Ch_2_
13	17.84	7,8-Didehydro-3-methylprostreptovaricin	654.333	M + H	C36H47NO10	Ch_1_
14	19.99	1-(2-(((Z)-2-(dimethylamino)-1-hydroxy-3-phenylpropylidene) amino)-3-methylpentanoyl)-3-(4-((E)-2-(((1Z,2E)-1-hydroxy-3-phenylallylidene) amino) vinyl) phenoxy) pyrrolidine-2-carbimidic acid	666.3721	M + H	C39H47N5O5	Ch_1_
15	20.47	N-isopentyltridecanamide	284.295	M + H	C18H37NO	Ch_2_
16	22.42	2-Methyl-3-[(2R)-1-methylpyrrolidin-2-yl] pyridine	177.1385	M + H	C11H16N2	Ch_1_ and Ch_2_
17	23.08	3''''-Adenylylspectinomycin	684.204	M + Na	C24H36N7O13P	Ch_2_
18	23.29	PC(P-18:1(11Z)/P-16:0)	728.59	M + H	C42H82NO6P	Ch_2_
19	23.31	PC(o-18:1(9Z)/18:1(11Z))	772.616	M + H	C44H86NO7P	Ch_2_
20	23.5	1-hexadecyl-2-[(4Z,7Z,10Z,13Z,16Z,19Z)-docosahexaenoyl]-sn-glycero-3-phosphocholine	792.5982	M + H	C46H82NO7P	Ch_2_
21	24.74	N-(1,3-dihydroxytetradec-4-en-2-yl)octadecanamide	532.474	M + Na	C32H63NO3	Ch_2_
22	25.3	3-(octadec-9-enoyloxy)-2-(octadeca-9,12,15-trienoyloxy)propyl icosa-9,14-dienoate	945.7366	M + K	C59H102O6	Ch_1_
23	25.57	TG(15:0/22:6(4Z,7Z,10Z,13Z,16Z,19Z)/22:6(4Z,7Z,10Z,13Z,16Z,19Z))	959.716	M + Na	C62H96O6	Ch_2_
24	25.92	menaquinone-11	959.7177	M + K	C66H96O2	Ch_1_
25	26.63	2-((tert-butoxy(hydroxyl)methylene)amino)-N-(1-((4-guanidinobutyl)imino)-1-hydroxy-4-methylpentan-2-yl)-4-methylpentanimidic acid	457.3494	M + H	C22H44N6O4	Ch_2_
26	29.49	N-(2-(tetracos-15-enoylthio)ethyl)acrylimidic acid	502.3712	M + Na	C29H53NO2S	Ch_1_
27	30.42	Phosphatidylethanolamine (24:1/24:0)	936.7493	M + Na	C53H104NO8P	Ch_2_

## Conclusion

The present study demonstrates that Egyptian immature Citrus honey contains species of *Lactobacilli* with varying antimicrobial activity against human pathogens. Although *lactobacilli* strains in this study have potential anti- lung cancer effect, the safety study of this isolates is needed further in-depth exploration to be applied for cancer treatment effectively. Results suggest that stx1 stx2 and eae down regulation may be related to secondary metabolites produced by *Levilactobacillus brevis* Ch_1_. A rapid and sensitive technique based on HPLC–Q-TOF–MS/MS was assessed for the separation and identification of chemical constituents and in vivo metabolites of *lactobacillus* stains. In a 30-min analysis, 27 compounds were identified or tentatively characterized with antifungal capabilities. It’s worth noting that the presence of LAB in honey may be an important factor to consider when selecting honey for pharmacological use. health care, biocontrol agent, and food product applications as well. Further work targeting their technological characteristics and viability in the fermented food matrix is needed.
